# The First Harmonic of Radial Pulse as an Early Predictor of Silent Coronary Artery Disease and Adverse Cardiac Events in Type 2 Diabetic Patients

**DOI:** 10.1155/2018/5128626

**Published:** 2018-10-23

**Authors:** Chi-Wei Chang, Kuo-meng Liao, Yi-Ting Chang, Sheng-Hung Wang, Ying-chun Chen, Gin-Chung Wang

**Affiliations:** ^1^Graduate Institute of Biomedical Electronics and Bioinformatics, National Taiwan University, Taipei, Taiwan; ^2^Division of Endocrinology and Metabolism of Zhongxiao Branch of Taipei City Hospital, Taipei, Taiwan; ^3^Biostatistics, Johns Hopkins Bloomberg School of Public Health, Baltimore, USA; ^4^Metal Industries Research & Development Centre, Kaohsiung, Taiwan; ^5^JinMu Health Technology, Taipei, Taiwan

## Abstract

**Background:**

It has been reported that harmonics of radial pulse is related to coronary artery disease (CAD) in patients with type 2 diabetes mellitus (T2DM). It is still unclear whether or not the first harmonics of the radial pulse spectrum is an early independent predictor of silent coronary artery disease (SCAD) and adverse cardiac events (ACE).

**Objectives:**

To measure the risk of SCAD in patients with T2DM and also to survey whether or not an increment of the first harmonic (*C*1) of the radial pulse increases ACE.

**Methods:**

1968 asymptomatic individuals with T2DM underwent radial pulse wave measurement. First harmonic of the radial pressure wave, *C*1, was calculated. Next, the new occurrence of ACE and the new symptoms and signs of coronary artery disease were recorded. The follow-up period lasted for 14.7 ± 3.5 months.

**Results:**

Out of 1968 asymptomatic individuals with T2DM, ACE was detected in 239 (12%) of them during the follow-up period. The logrank test demonstrated that the cumulative incidence of ACE in patients with *C*1 above 0.96 was greater than that in those patients with *C*1 below 0.89 (*P* < 0.01). By comparing the data of patients with *C*1 smaller than the first quartile and the patients with *C*1 greater than the third quartile, the hazard ratios were listed as follows: ACE (hazard ratio, 2.29; 95% CI, 1.55–3.37), heart failure (hazard ratio, 2.22; 95% CI, 1.21–4.09), myocardial infarction (hazard ratio, 2.44; 95% CI, 1.51–3.93), left ventricular dysfunction (Hazard ratio, 2.01; 95% CI, 0.86–4.70), and new symptoms and signs for coronary artery disease (hazard ratio, 2.03; 95% CI, 1.45–2.84). As *C*1 increased, the risk for composite ACE (*P* < 0.001 for trend) and for coronary disease (*P* < 0.001 for trend) also increased. The hazard ratio and trend for cardiovascular-cause mortality were not significant.

**Conclusions:**

This study showed that *C*1 of the radial pulse wave is correlated with cardiovascular events. Survival analysis showed that *C*1 value is an independent predictor of ACE and SCAD in asymptomatic patients with T2DM. Thus, screening for the first harmonic of the radial pulse may improve the risk stratification of cardiac events and SCAD in asymptomatic patients although they had no history of coronary artery disease or angina-related symptom.

## 1. Introduction

Coronary artery disease (CAD) is one of the leading causes of death and contributes to the adverse cardiovascular events. Compared with nondiabetics, patients with type 2 diabetes mellitus (T2DM) have 2- to 4-fold increase risk of CAD [1] and are more often without history and symptoms of coronary artery disease until the onset of myocardial infarction or sudden cardiac death [2]. An asymptomatic patient with T2DM has been shown 20–35% prevalence of silent CAD (SCAD) [3, 4], which could lead to myocardial ischemia, adverse cardiac events (ACE), and a poor prognosis outcome [5–9]. Hence, it is essential to have early predictors to screen the high-risk asymptomatic diabetic patients, to give a risk assessment of SCAD and ACE. Thus, diabetic patients with SCAD may benefit from screening and further medical intervention to prevent sudden cardiac death or adverse cardiovascular events.

Several studies made an effort on the identification of those diabetic patients with SCAD [10], using coronary artery calcium imaging or stress radionuclide myocardial perfusion imaging (MPI) [11–14]. However, the risk of the stress test and the radiation dose in those images makes the screening of diabetic patients with SCAD remain controversial. European society of cardiology guideline suggested class II to class III recommendation and was not used in patients with a low range of intermediate of pretest probability for CAD [15]. Therefore, the patients with diabetes and clinically at high risk of SCAD need to be further defined and investigated before calcium imaging score, MPI, or invasive coronary angiography (ICA) is performed.

The emerging technical method to screen SCAD is using the harmonic index in arterial pulse spectrum analysis. In the 1970s, medical researchers introduced harmonic analysis to convert pressure pulses and flow pulses into numerical Fourier series [16]. Harmonics is one of the complete quantitative descriptions of periodic pulse waveforms [17]. Initially, the harmonic analysis was applied to the transfer function between the aortic pulse wave and the radial pulse wave [18]. In recent decades, many studies have used harmonic analysis to assess vascular sclerosis [19], aortic occlusion [20], aging effects [21], vasodilator drugs [22], and heart diseases [23, 24]. Chen et al. first found that the specific characteristic of radial pulse spectrum changed from the resting state to the onset of acute, uncomplicated myocardial infarction state, and gradually shifted to other resting characteristics a week after the surgery [25]. A study suggests that harmonic characteristics may be an adjunct method for identifying patients who require intensive treatment or revascularization [26, 27].

In addition, the important relationship between the first harmonic of the radial pulse wave and cardiac risk has been studied recently. Wang et al. demonstrated that the first harmonics of the radial pulse increased with age [21]. The cross-sectional cohort study showed that the first, second, and third harmonics of the radial pulse correlated with the ischemic heart symptoms [28]. The population mean of the first harmonic is also higher in asymptomatic diabetic patients with more than 5% of ischemic myocardium, compared with those without significant myocardial perfusion defects (*P* < 0.05) [29]. Since the first harmonic is associated with the aging process and with the signs and symptoms of myocardial ischemia, the next issue is whether the first harmonic of radial pulse is an independent predictor for SCAD and for adverse cardiac events (ACE). Therefore, this study aimed to statistically validate the degree of confidence that the first harmonic of radial pulse is associated with SCAD and ACE in a prospective cohort study, using survival analysis and Cox regression model.

## 2. Methods

### 2.1. Patients

The present population included 1158 men and 810 women who had a record of T2DM and have already entered a diabetes management program in the Division of Endocrinology & Metabolism of Zhongxiao Branch of Taipei City Hospital. In order to investigate the asymptomatic patients, the key exclusion criteria were listed as follows: (1) angina pectoris or angina equivalent symptoms; (2) any stress test or coronary angiography before the enrollment; (3) shortness of breath either at rest or on exertion; (4) history of myocardial infarction, heart failure, or coronary revascularization; (5) electrocardiographic evidence of Q-wave abnormality, ischemic ST-segment, and T-wave abnormality; (6) clinically significant valvular heart disease or cardiomyopathy. Subjects were also excluded if the radial pulse wave measurement could not be carried out due to the severer diseases or acute symptoms such as end-stage renal disease or liver disease. A total of 1968 patients have been enrolled in the study between January 2017 and May 2018 in Taiwan. After the assessment of the first harmonic amplitude of the radial pulse wave at baseline, patients were followed for a mean of 14.7 ± 3.5 months. The study was approved by the Institutional Review Board of Taipei City Hospital (IRB number: ISRCTN14306167). Then, both oral and written information about the study will be given to the enrolled patients. All participants signed for their consent.

### 2.2. Study Design

The medical history and physical examination report were retrieved to determine the patient demographic data, the status of T2DM, and other clinical variables. Readings of radial pulse and harmonic analysis were taken in 1968 participants at baseline with a longest follow-up period that lasted for 16 months after the radial pulse measurement. Myocardial infarction, heart failure, and cardiovascular death were also documented.

The baseline of the first harmonics of the radial pulse was carried out at the beginning of the study. Each subject was required to lie down in a supine position and rest for 5 minutes before the measurement. The pulse pressure was then obtained on the left-hand radial artery with a pulse wave analyzer TD01C (MII-ANN Technology, Taiwan). TD01C has been proved its intrinsic reliability using an artificial pulse generator [30]. The intraobserver and interobserver reliabilities of TD01C were also manifested in the clinical study [31]. The successive pressure pulses were acquired during the 12-second period. The pressure pulse data were recorded with a sampling rate of 400 data points per second. These data were then transformed into harmonic components (Cn) and phase angle (Pn) using Fourier transform. *C*1 is defined by the following equation:

(1) $$\begin{array}{c} C1=\frac{A_{1}}{A_{0}}, \end{array}$$

where *A*_0_ is the mean value of pulse wave, and *A*_1_ is the 1st amplitude coefficient of Fourier series of the radial pulse wave. The mean value of *C*1 calculated from all pulses was used as a representative harmonic amplitude value within one measurement. After the assessment of radial pulse spectrum, the blood pressure and heart rate were assessed by an automatic blood pressure monitor (HBP-9020, Omron, Japan) according to the instruction manual. The assessment was performed by a well-trained operator, without the doctors and nurse presented, to avoid the white coat effect.

### 2.3. Outcomes

The patients entering the diabetes management program will visit the research hospital every 4–6 months to conduct the clinical evaluation of cardiac risk, based on the risk factor assessment or cardiovascular events. The study used the risk factor evaluation to select the patients who need a further cardiac stress test such as SPECT myocardial perfusion image, or stress electrocardiogram (ECG), mostly following the guidelines [24, 25]. The candidates for stress test are the patients with two or more following risk factors: (i) lipid disorders (total cholesterol ≧ 240 mg/dl, low-density lipoprotein (LDL) ≧ 160 mg/dl, or high-density lipoprotein (HDL) ≦ 35 mg/dl), (ii) blood pressure > 140/90 mmHg, (iii) family history of premature coronary heart disease, (iv) age ≧ 70 years, (v) newly discovered resting ECG abnormalities, (vi) ankle-brachial index ≦ 0.9, and (vii) albumin creatinine ratio ≧ 30 mg/g. After stress testing, revascularization will be considered in patients with moderate to severe myocardial ischemia (summed stress score ≥ 9) or abnormal stress ECG.

The primary study outcome was a composite of myocardial infarction, heart failure, or cardiovascular mortality. The secondary outcome was a composite of left ventricular dysfunction (LVEF ≤ 50%), and the new occurrence of symptoms and signs of coronary artery disease, which was defined by the related symptoms and signs of CAD diagnosed by the cardiology physicians.

Myocardial infarction was diagnosed by a cardiologist physician following the consensus guideline for Joint European Society of Cardiology/American College of Cardiology Committee [32] and the definition of World Health Organization, including a combination of at least two of three characteristics as follows:

1. Enzyme rise: an increased value for cardiac troponin, exceeding the value of the 99th percentile of a reference control group, reflecting myocardial cell death.
2. Abnormal electrocardiography: myocardial ischemia (ST-T segment changes) or evidence of loss of electrically functioning cardiac tissue (Q waves)
3. The angina-related symptoms.

Heart failure was diagnosed by a cardiologist physician, including identification of a structural and functional abnormality, following the New York Heart Association (NYHA) Functional Classification and the guideline of American College of Cardiology [33, 34].

The signs of coronary artery disease included the newly diagnosed single coronary stenosis (>70%), multicoronary stenoses (>50% stenosis for at least two major coronary vessels), and more than mild myocardial ischemia using semiquantitative 20-segment analysis of myocardial perfusion single-photon emission computed tomography (GE Infinia, USA). Each of these outcomes was also analyzed separately. Source data were derived from the database of the diabetes management program in Zhongxiao Branch of Taipei City Hospital and were verified by independent monitors.

### 2.4. Statistical Analysis

**Figure 1:**
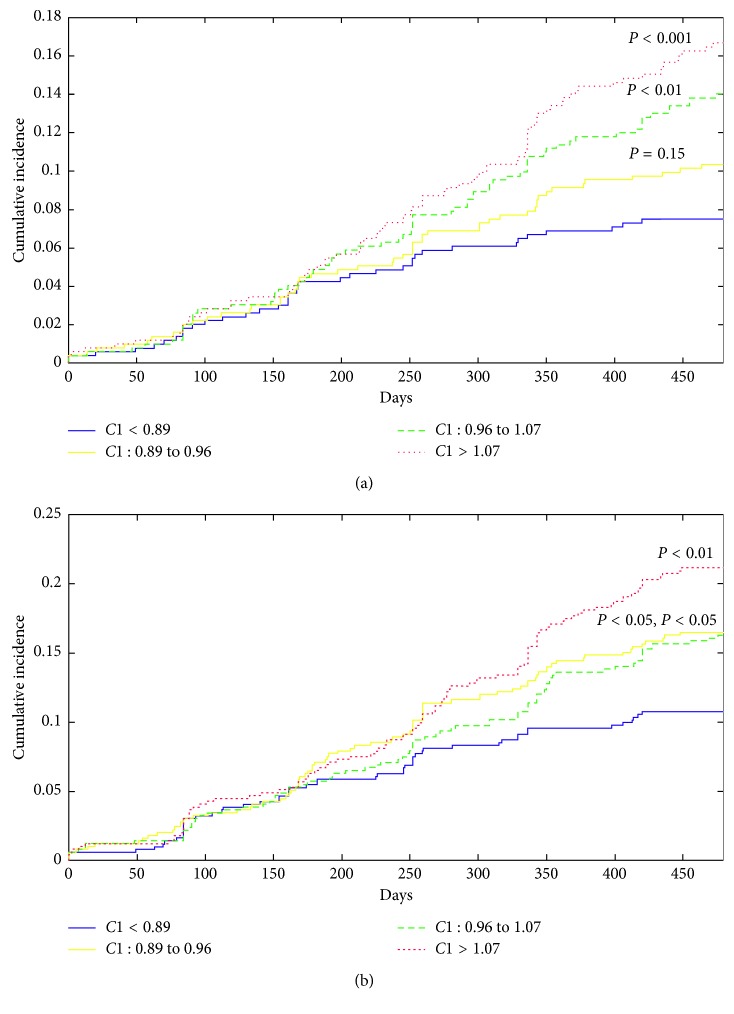
Kaplan–Meier event rates of the (a) primary and (b) secondary composite outcomes. Primary composite endpoints combined the adverse heart events, including new onset of heart failure, myocardial infarction, and cardiovascular mortality. Secondary composite endpoints combined the left ventricular dysfunction (LVEF < 50%) and the symptoms and signs of coronary artery disease (*n*=1968); *P* values were the result of the logrank test. The reference group for logrank test is the first quartile of *C*1 (<0.89).

The study was designed to follow the patients for 16 months. All the subjects were categorized by four quartiles of *C*1 levels (<0.89, 0.89 to 0.96, 0.96 to 1.07, and >1.07), in which Cox proportional hazards model were adopted to compare hazard ratios of clinical events among quartile groups with reference to the first quartile (*C*1 < 0.89). The unadjusted hazard ratio and 95% confidence intervals of all outcomes in each quartile were recorded. Tests for linear trend across quartiles were examined using Cox regression analysis. Linear trend tests were assessed both before and after adjusting for age, sex, smoking, systolic pressure, diastolic blood pressure, dyslipidemia, and Hba1c. The curves of Kaplan–Meier for primary and secondary composite endpoints are plotted in Figure 1 according to the quartile levels of *C*1. In order to determine if *C*1 has a significant effect on the risk of adverse heart events and loss of cardiac function, the logrank test was performed. Matlab version 9.2 (MathWorks Inc, USA) was used to conduct all statistical analyses.

## 3. Results

The baseline clinical characteristics of 1968 asymptomatic patients with T2DM were shown in Table 1. The mean age of participants was 62 ± 12 years, in which 58.8% of the participants were male. The mean systolic, diastolic blood pressure, and the Hba1c level were 128 ± 12 mmHg, 75 ± 31 mmHg, and 7.0 ± 1.1%, respectively. Kaplan–Meier curve showed the primary and secondary composite endpoints of four groups, within 16 months follow-up. There was a graded relationship between the baseline *C*1 and risk of ACE. The cumulative incidence of primary composite endpoint increased from 7.5% to 10.4% to 14% to 16.7% according to four quartiles of *C*1 levels. This relationship extended into the heart function loss and CAD. The cumulative incidence of secondary composite endpoint increased from 10.8% to 16.5% to 16.3% to 24.1% according to the same four quartiles of *C*1 levels.

The listed incidence and the hazard ratios are written in Table 2. Compared with the first quartile (*C*1 < 0.89), the hazard ratio of primary composite endpoint for each quartile was 1.39 (95% CI, 0.91–2.12) for *C*1 of 0.89 to 0.96, 1.90 (95% CI, 1.28–2.84) for *C*1 of 0.96 to 1.07, and 2.29 (95% CI, 1.55–3.37) for *C*1 > 1.07. The linear trends were significant for major adverse cardiac events before and after controlling for age, sex, smoking, systolic pressure, diastolic blood pressure, dyslipidemia, and Hba1c (*P* < 0.001).

Similar findings were also found in the hazard ratio of the secondary composite endpoint for each quartile: Compared with the first quartile (*C*1 < 0.89), the hazard ratio of secondary composite endpoint for each quartile were 1.56 (95% CI, 1.11–2.21) for *C*1 of 0.89 to 0.96, 1.53 (95% CI, 1.08–2.17) for *C*1 of 0.96 to 1.07, and 2.04 (95% CI, 1.46–2.83) for *C*1 > 1.07. The linear trends were significant for the symptoms and signs of CAD before and after controlling for age, sex, smoking, systolic pressure, diastolic blood pressure, dyslipidemia, and Hba1c (*P* < 0.001).

## 4. Discussion

The prevalence of SCAD in patients with T2DM varied from 10% to over 50% according to different study settings [35]. The diabetic patients with SCAD showed higher incidences of cardiovascular events and deaths compared with no-SCAD diabetic patients using MPI [36–38], which indicated the need to identify the diabetic patients at high risk of SCAD. In asymptomatic patients with T2DM, whether carrying out the further investigation of CAD or not is a dilemma between the increasing risk of adverse heart disease and the increasing risk due to radiation exposure of coronary artery calcium imaging and due to the stress testing such as myocardial perfusion single-photon emission computed tomography. The European society of cardiology guideline recommends not using those testing in patients with a low range of intermediate of pretest probability for CAD [15]. Therefore, without any history of CAD and angina-related symptoms, a proportion of the diabetic patients with SCAD were unrecognized until the onset of ACE [3, 4, 39].

Since diabetes increases the cardiac risk, the need for new risk markers for SCAD exists. The patients with T2DM and at high risk of SCAD need to be further identified before calcium imaging score, MPI, or invasive coronary angiography (ICA) is performed. The ambulatory ECG has been demonstrated useful in predicting CAD and future cardiovascular event in asymptomatic diabetic patients [40]. However, ambulatory ECG had two limitations. First, ambulatory ECG was limited due to its lack of specificity, ranging from 50%–60% [41]. Second, there is no clear evidence for ambulatory ECG to provide reliable information about myocardial ischemia in asymptomatic patients, especially those without known CAD [42]. Also, Zellweger et al. combined several traditional risk factors of CAD to screen the asymptomatic high-risk patients with diabetes and to predict the abnormal score of MPI [43]. Ankle-brachial index (ABI) has been proved to be associated with CAD [44–47], and low ABI was investigated as a predictor of CAD [48, 49]. Diabetic patients with microalbuminuria (albumin-to-creatinine ratio: 30–300 mg/g) had also been confirmed with increased risk of CAD [50]. ABI measured a ratio of ankle systolic blood pressure to brachial systolic pressure and assessed the presence of occlusive peripheral arterial disease and generalized atherosclerosis [48, 51]. Both low ABI [52] and microalbuminuria [53–55] have been manifested in the detection of silent myocardial ischemia symptom.

Recently, the cross-sectional study screened the performance of ABI, microalbuminuria, and harmonics of the radial pulse to stratify the risk of SCAD and severe silent myocardial ischemia in patients with T2DM and had at least one traditional risk factor. The harmonics of the radial pulse (ORs, 2.36–4.46), ABI (ORs, 2.24–6.90), and microalbuminuria (ORs, 0.5–1.70) showed different abilities to identify the patients at high risk of CAD [26]. Combining those risk markers using majority votes, the diagnostic performance for silent myocardial ischemia (area under ROC: 0.74) and for SCAD (Area under ROC: 0.82) was greatly improved compared with traditional ASCVD score in asymptomatic patients with T2DM [26].

This study, using noninvasive radial pulse waveform and harmonic analysis, further surveyed the predictive values of the first harmonic index for the heart disease, from the symptoms and signs of CAD and the left ventricular dysfunction to the ACE (myocardial infarction, heart failure, and cardiovascular death) in a prospective cohort study. The report demonstrated that the first harmonic of radial pulse is an independent risk factor for the symptoms and signs of CAD, the ventricular dysfunction, myocardial infarction, and heart failure. The possible mechanism could be deduced from the resonance effect of the whole arterial system, which was proposed and validated by Wang et al. [56]. The further study manifested that function of the organ can be reflected on the radial pulse spectrum [57].

The amplitude and phase of harmonic phases can be affected by arterial stiffness and the loading condition in the phantom study of the arterial system [58–60]. Consistent with the phantom study, several clinical tests also manifested that the arterial waveform revealed the status of arterial stiffness [61, 62] and the coronary artery perfusion [25]. Two clinical studies also found that augmentation index and augmented pressure, the calculated index of the radial pulse waveform, are independent risk markers for premature coronary artery disease [63] and for major adverse cardiac events [64] in a prospective study. Furthermore, Kingwell et al. found that the large arterial stiffness affected the arterial waveform and is associated with a reduction in myocardial contractile reserve and with myocardial ischemia symptom [65]. The above clinical study described the interactions among arterial pressure waveform, arterial stiffness, and cardiovascular events.

Wang's research shows that the first harmonic of the radial pulse wave increases with age and the augmentation index and suggests that atherosclerosis may play an important role in this age-related change of harmonics [21]. An animal study supports this concept and demonstrates that aortic stiffness in aged mice results in a significant increase in first harmonic impedance [19]. In a mice study, Segers et al. also found that the increase in cardiac afterload leads to the increase in the first harmonic impedance and the increasing need of stroke work in each heart pumping [20]. In other words, the first harmonic impedance increases along with atherosclerosis or increasing afterload, resulting in the increasing burden of heart muscle. Furthermore, an increase in the first harmonic impedance results in an increase in the harmonic amplitude of arterial pulse for the same desired stroke volume. The more stroke work increases the oxygen consumption of myocardium and may increase the risk of myocardial ischemia and heart dysfunction. Therefore, this mechanism might also explain why the first harmonic of the radial artery is larger in patients with ischemic myocardium compared with controlled patients [29]. A cardiac research study supports this concept and shows that patients with heart failure had significantly higher first harmonic aortic impedance [23]. This report also showed a consistent result that the patients with a higher level of *C*1 had a larger risk of left ventricular dysfunction, myocardial infarction, and heart failure (Table 2). However, there are many gaps among the current knowledge about the arterial pulse waveform and mechanisms of how the hemodynamic status of the ventricular-arterial system affect the pulse waveform. The further investigation is warranted to uncover the detailed relationship between the radial pulse spectrum and the risk of heart disease.

This study has some limitations. First, the radial pulse measurements were performed at baseline when enrolled patients visited the study area for the first time. Therefore, it is necessary to determine the reliabilities of the TD01C measuring instrument for research purposes. In the previous clinical study for intraobserver reliability, the population mean and standard error of the difference of *C*1 values between successive measurements was −0.006 ± 0.006 [31]. In the interobserver reliability study, the population mean, and standard error of the difference of *C*1 values between successive measurements was −0.002 ± 0.008 [31]. This showed the contribution of operator-induced bias, and variation is relatively low compared with the different levels of *C*1 values between quartile groups. The reliability testing also demonstrated that there are no significant white coat effects for a pulse spectrum. The population mean of the difference of *C*1 values between successive measurements within 5 minutes was close to zero. In addition, another clinical study showed that there was no significant difference in pulse spectra within 2 hours of five measurements (one measurement every 30 minutes), if only warm water was consumed before the first measurement [66]. For each same subject, there was no significant change in the pulse spectrum between the different measurements over the different weeks [66]. This indicated that following the standard protocol and consistent clinical procedures, the pulse wave measurements are reliable. Therefore, the white coat effects may have only a slight impact on the pulse spectrum since the subjects were well informed about the pulse spectrum measurement. Then, the results were based on a short-term prospective cohort study. The independency of *C*1 as a cardiac risk marker only depends on about 500 events within 16 months of follow-up. More clinical studies are needed to confirm the independence of *C*1 as a risk marker in a more general population. We have applied an extended study for 3 years of follow-up to investigate further the relationships among harmonics, traditional risk factors, and adverse cardiac events.

## 5. Conclusion

This study showed that the first harmonics amplitude of the radial pulse, *C*1, is associated with cardiovascular events. The risk of primary and secondary composite endpoint was all increased with increasing quartile of graded *C*1 levels. The results of the survival analysis demonstrated that the *C*1 value is an independent predictor of ACE in asymptomatic patients with T2DM. Hence, periodic screening for radial pulse spectrum may improve the identification of type 2 diabetic patients at high risk of SCAD and confronting ACE in the future, who may need a further investigation or preventive treatment to reduce the cardiac risk.

## Figures and Tables

**Table 1:** Baseline clinical characteristics of the asymptomatic patients with type 2 diabetes.

Clinical characteristics	Value (*n*=1968)
Male (%)	58.8
Age (year)	61.6 ± 12.5
BMI (kg/m^2^)	27.2 ± 4.6
Waist circumference (cm)	93 ± 12
Smoke (%)	15.2
SBP (mmHg)	128 ± 12
DBP (mmHg)	74 ± 8
PP (mmHg)	53 ± 10
HbA1C (%)	7.0 ± 1.1
EGFR (mL/min/1.73 m^2^)	87 ± 34
LDL (mg/dl)	86 ± 28
HDL (mg/dl)	50 ± 16
TC (mg/dl)	160 ± 34
TG (mg/dl)	129 ± 81
Heart rate (beats/min)	73 ± 11
Duration of diabetes (years)	10 ± 7

SBP = systolic blood pressure, DBP = diastolic blood pressure, PP = pulse pressure, Hba1c = glycated hemoglobin, LDL = low-density lipoprotein cholesterol, HDL = high-density lipoprotein cholesterol, TC = total cholesterol, TG = triglycerides, and EGFR = estimated glomerular filtration rate.

**Table 2:** Quartile of the first harmonic of the radial pulse wave as a risk for primary and secondary composite heart endpoints in 1968 type 2 diabetic patients without CAD history and angina-related symptoms. Primary composite endpoints combined the adverse heart events, including new onset of heart failure, myocardial infarction, and cardiovascular-cause mortality. Secondary composite endpoints combined the new onset of left ventricular dysfunction (LVEF < 50%) and the new onset of symptoms and signs of coronary artery disease.

Endpoint	*C*1, first harmonic of the radial pulse wave					
	<0.89	0.89 to 0.96	0.96 to 1.07	>1.07	*P* for trend	*P* for trend^#^
*Primary composite endpoints*						
Patients, *n* (%)	37 (7.5%)	51 (10.4%)	69 (14.0%)	82 (16.7%)		
Hazard ratio (95% CI)	1	1.39 (0.91–2.12)	1.90 (1.28–2.84)	2.29 (1.55–3.37)	<0.001	<0.001

Heart failure						
Patients, *n* (%)	15 (3.0%)	17 (3.5%)	24 (4.9%)	33 (6.7%)		
Hazard ratio (95% CI)	1	1.13 (0.57–2.27)	1.61 (0.84–3.06)	2.22 (1.21–4.09)	<0.01	<0.05

Myocardial infarction						
Patients, *n* (%)	24 (4.9%)	37 (7.5%)	49 (10.0%)	57 (11.6%)		
Hazard ratio (95% CI)	1	1.56 (0.93–2.60)	2.08 (1.28–3.39)	2.44 (1.51–3.93)	<0.001	<0.001

Cardiovascular-cause mortality						
Patients, *n* (%)	2 (0.4%)	0 (0%)	2 (0.4%)	2 (0.4%)		
Hazard ratio (95% CI)	1		1.00 (0.14–7.09)	1.00 (0.14–7.09)	NS	NS

*Secondary composite endpoints*						
Patients, *n* (%)	53 (10.8%)	81 (16.5%)	80 (16.3%)	104 (21.1%)		
Hazard ratio (95% CI)	1	1.56 (1.11–2.21)	1.53 (1.08–2.17)	2.04 (1.46–2.83)	<0.001	<0.001

Left ventricular dysfunction (LVEF < 50%)						
Patients, *n* (%)	8 (1.6%)	11 (2.2%)	16 (3.3%)	16 (3.3%)		
Hazard ratio (95% CI)	1	1.38 (0.56–3.44)	2.01 (0.86–4.71)	2.01 (0.86–4.70)	<0.1	<0.05

Symptoms and signs of coronary artery disease						
Patients, *n* (%)	51 (10.4%)	75 (15.2%)	75 (15.2%)	100 (20.3%)		
Hazard ratio (95% CI)	1	1.49 (1.05–2.13)	1.49 (1.04–2.12)	2.03 (1.45–2.84)	<0.001	<0.001

The reference group for hazard ratio is the first quartile of *C*1 (<0.89). NS = nonsignificant. ^*#*^*P* for trend controlling for age, sex, smoke, systolic and diastolic blood pressure, dyslipidemia, and Hba1c.

## Data Availability

The data used to support the findings of this study are available from the corresponding author upon request.
